# Sound quality impacts dogs’ ability to recognize and respond to playback words

**DOI:** 10.1038/s41598-025-96824-8

**Published:** 2025-04-28

**Authors:** Fumi Higaki, Tamás Faragó, Ákos Pogány, Ádám Miklósi, Claudia Fugazza

**Affiliations:** 1 Department of Ethology, Eötvös Loránd University, Pázmány P. s 1c, 6th Floor, Budapest, 1117 Hungary; 2 BARKS Lab, Department of Ethology, Eötvös Loránd University, Budapest, Hungary; 3 ELTE-HUN REN NAP Comparative Ethology Research Group, Budapest, Hungary; 4 MTA-ELTE Comparative Ethology Research Group, Budapest, Hungary

**Keywords:** Vocal recognition, Audio quality, Sound processing equipment, Augmentative interspecies communication (AIC), Dog, Psychology, Human behaviour

## Abstract

Humans show a remarkable ability to recognize degraded speech, but they struggle as degradation becomes more severe. Research on dogs, a species naturally exposed to human language, has suggested that also in this species, the level of degradation may negatively correlate with familiar speech recognition. The quality of sound playback equipment is crucial in canine research to ensure accurate reproduction, directly impacting dogs’ receptivity. In citizen science, availability, operability, and affordability are also key. However, the varying level of sound degradation of commercially available devices may impact dogs’ recognition of the played-back sounds. This study evaluates how different audio devices affect the response to human speech in dogs. Study 1 tested dogs’ responses to trained verbal action cues delivered via Augmentative Interspecies Communication (AIC) buttons, loudspeakers, and direct human speech. Study 2 tested Gifted Word Learner (GWL) dogs’ accuracy in retrieving named toys using the same devices. Study 3 investigated whether GWL dogs could learn new toy names through recorded voice played via loudspeakers and generalize these to direct human speech. Results consistently showed that dogs’ responses to verbal cues were significantly affected by the device type, with the best performance observed for direct human speech and the lowest performance for AIC buttons. Our findings show that the level of sound degradation significantly affects dogs’ recognition of recorded speech and suggest that current commercially available AIC devices are not recommended for testing effective recognition of the recorded words.

**Supplementary Information:**

The online version contains supplementary material available at 10.1038/s41598-025-96824-8.

## Introduction

Research has extensively examined speech recognition in adverse conditions^1^, and humans appear to have a remarkable ability to comprehend speech under various conditions that significantly alter the sounds they hear. Findings indicate that even toddlers and young children can recognize degraded speech – more specifically, vocoded speech^2^ - to some extent^3^. Comprehension improves with increased vocabulary size and cognitive maturity; however, listeners struggle to recognize the speech with heavy degradation^4^.

Some research has explored whether this ability is uniquely human or relies on general cognitive processes that may also exist in other species. Dogs, thanks to their natural development in the human (linguistic) environment, are an ideal candidate species for studies investigating their ability to recognize familiar degraded speech. Previous studies suggest that they recognize their names even in the presence of relatively difficult levels of background noise^5^ and also when the speech signal is degraded to some extent^6^. On the other hand, studies using dogs’ responses to tape-recorded action commands report a significant decline in dogs’ performance, suggesting that dogs may not find the recorded commands identifiable due to the lack of certain glottal source signal features, resulting in differences in frequency composition, harmonics, and resonance compared to human-generated cues^7^.

Digital sound processing has brought significant advancements in how we capture, manipulate, store, and reproduce sound. However, commercially available audio communication products often involve trade-offs to meet consumer needs, including compromises in microphone and speaker quality for affordability and compressed format quality for smaller file sizes. These limitations can result in missing frequencies and reduced audio fidelity. Although this degradation may not be noticeable in typical human conversational speech^8^, these compromises may not align with the perceptual needs of other species, such as dogs.

Humans can typically hear frequencies ranging from 20 Hz to 20,000 Hz^9^, with key speech frequencies falling between 300 Hz and 3,400 Hz^10^. In comparison, dogs hear frequencies between 65 Hz and 45,000 Hz, showing greater sensitivity to higher, even ultrasonic frequencies. Playback equipment is almost exclusively optimized for human auditory perception, with frequency responses typically designed to capture sounds up to 20 kHz.

The use of audio recordings in research experiments provides the benefit of consistency and repeatability. However, several factors such as microphone sensitivity, file formats applying lossy compression of sound data^11^, and speaker frequency profiles play a critical role in determining the quality for both recordings and playback required to serve the purpose.

In canine research, recorded audio has been employed in various studies to explore how dogs respond to human voices. Tape recordings often suffer from inherent noise, such as tape hiss, yet they have been employed in various studies. For instance, tape recordings were used to investigate dogs’ responses to recorded action cues with modified phonemes^12,13^. Rossi and Ades^14^ used a keyboard with tape-recorded voices to explore whether a dog could learn to press specific keys to obtain specific rewards. Recent studies have successfully employed digital recordings to investigate various aspects of human voice identity processing^15^, pet-directed speech^16^, and digital recordings are typically used in fMRI studies to examine brain activation upon hearing human voices^15,17–19^.

These audio technologies have facilitated studies on canine abilities to process human voices, whereas the consumer market has taken a different approach to leveraging technology for interspecies communication. Recently, Augmentative Interspecies Communication (AIC) devices have become popular among dog owners, driven largely by social media trends and popular books^20^. The “talking button” products are marketed as “*tools in teaching human language to animals in order to allow them to ‘speak their minds’* ”^21^ and are gaining widespread use among dog owners. This surging public interest calls for scientific scrutiny, as it has been claimed to yield the potential to advance knowledge of animal behavior, cognition, welfare, and even two-way communication with animals^22^.

Recently, research on interspecies communication between dogs and humans using AIC soundboard devices has emerged. Utilizing a citizen science approach, nearly 2,000 participants from 47 countries^23^ have enrolled in this initiative. One of their latest studies investigated how soundboard-trained dogs responded to trained AIC button pressing^24^. The findings showed that dogs associated two out of three tested button pressings with systematically presented outcomes. As the experiment did not include conditions in which the button positions were varied and considering that dogs typically prioritize spatial information^25–27^, dogs might have learned to respond to button pressing actions in specific positions rather than (or in addition to) the played-back sound. Moreover, it is possible that the dogs associated the specific sound played by the buttons to its outcome without recognizing its similarity with human spoken words. However, it is reasonable to assume that owners register their voices on the devices, believing that their dogs can recognize these sounds as if they were spoken. It remains uncertain how dogs perceive, recognize, and respond to human spoken words recorded and played back through different devices, and the growing use of AIC buttons urges research to answer this question. This information is also crucial for research employing recorded human speech.

Studies have also demonstrated that live social interaction plays a crucial role in infants’ language acquisition, whereas exposure to audiovisual recordings alone may be insufficient^28^. In dogs, the role of audiovisual recordings in learning new words remains unexplored. However, research has shown that social play interactions facilitate rapid object name learning in Gifted Word Learner (GWL) dogs, who possess the exceptional talent of being able to learn multiple object names, whereas typical dogs struggle with learning even a few^29,30^. Therefore, GWL dogs provide the opportunity to test speech degradation in a group of subjects with more experience and greater skills in understanding human words.

In this study, we test whether dogs can respond appropriately to familiar words played back using different devices with different levels of sound degradation, which arises from any alteration in the sound’s original characteristics when recorded and played back through different devices. This includes both the potential loss or attenuation of certain frequency ranges critical to dogs’ hearing and any unintended modification that may affect the harmonic and formant structure of the words. We hypothesized that human speech recognition by dogs is influenced by sound’s spectral degradation and that the more degraded words would result in poorer recognition, hence poorer responses from dogs compared to the less degraded sounds and to the words directly spoken by the owners. Additionally, because it is not known whether dogs rely on the owner’s mouth movement to recognize the different verbal cues, we tested this possibility by introducing conditions in which the owner moved his/her mouth as if saying the word when the corresponding sound was played through the devices.

In Study 1, we tested whether dogs recognize and respond to familiar human verbal cues for trained actions when emitted from commercially available AIC buttons, from a loudspeaker, and when these words are directly spoken by the owners. The tests were carried out with dogs trained to reliably perform actions on verbal cues spoken by their owners. Studies 2 and 3 exclusively tested GWL dogs because typical (T) dogs have a limited repertoire of trained action commands, whereas GWL dogs, with an average vocabulary of 29 toy labels^31^, allowed for greater variation in our tests. Additionally, GWL dogs can rapidly acquire the names of new toy^29^, enabling us to explore not only whether GWL dogs can recognize and respond to familiar words but also whether they can learn entirely new words under these more challenging conditions and generalize the learned (degraded) word to live human speech. In Study 2, using a method similar to Study 1, the same devices were used to test GWL dogs’ recognition and response to the names of toys from their acquired vocabulary. Study 3 investigated whether GWL dogs could learn new toy names through their owner’s recorded voice emitted by a loudspeaker and whether they could generalize those sounds to when the owner uttered the toy names live.

## General methods

### Materials

We utilized two audio recordings and playback systems:

1. FluentPet Classic Speak Up Button: This audio button device (‘button device’, henceforth), produced by CleverPet Inc., features a built-in microphone for recording and a speaker for playback. We selected this specific model for its distinction as “The Clearest, Loudest Button Available,” according to their product site (https://fluent.pet), and for its integrated capabilities to record and playback owners’ voices, which facilitates ease of use in the experimental setting.
2. Smartphone and JBL Go3 loudspeaker configuration: The participant’s smartphone (Apple iPhone, Google Pixel, Samsung Galaxy; the oldest model used was released in 2017) was connected to an external JBL Go3 loudspeaker for audio playback. The participants downloaded the Voice Record Pro app (version 4.0.0 by Dayana Networks Ltd) and used it to record their voice. All audio files were saved in WAV format and played through this app.

### Comparing the devices’ frequency outputs

As the manufacturer of the button device we used did not publish any technical specifications about the built-in audio parts (microphone and speaker frequency responses, used file format and resolution, loss of information), we ran a simple in-house benchmark test to explore the output quality differences between the used devices. We performed two recordings in a silent and soundproof lab. To match the recording capacity of the button device, we generated a 15-second long white noise sample using Audacity (version 3.7.0) and played this back while simultaneously recording with the button and a Pixel 6 smartphone, both placed 50 cm from the speaker (Magnat Monitor Supreme 102 speakers driven by JD labs Element III MK2 amplifier/DAC) on the floor. Next, we set up a Sennheiser shotgun microphone (ME-6X) on a stand placed where formerly the speaker was, linked with a Zoom H5 recorder and recorded the playbacks from the button and the smartphone, sequentially on the same recording without altering any settings. The smartphone was linked with the same JBL speaker used in the tests. Then, the recordings were transferred to a computer where using PRAAT^32^, we cut the re-recordings to match the original in length, generated spectrograms, and superimposed them on each other to reveal the spectral differences across the devices. (See Supplementary Table 1 for the audio files.)

Results of the comparison of the devices’ frequency outputs.

**Fig. 1:**
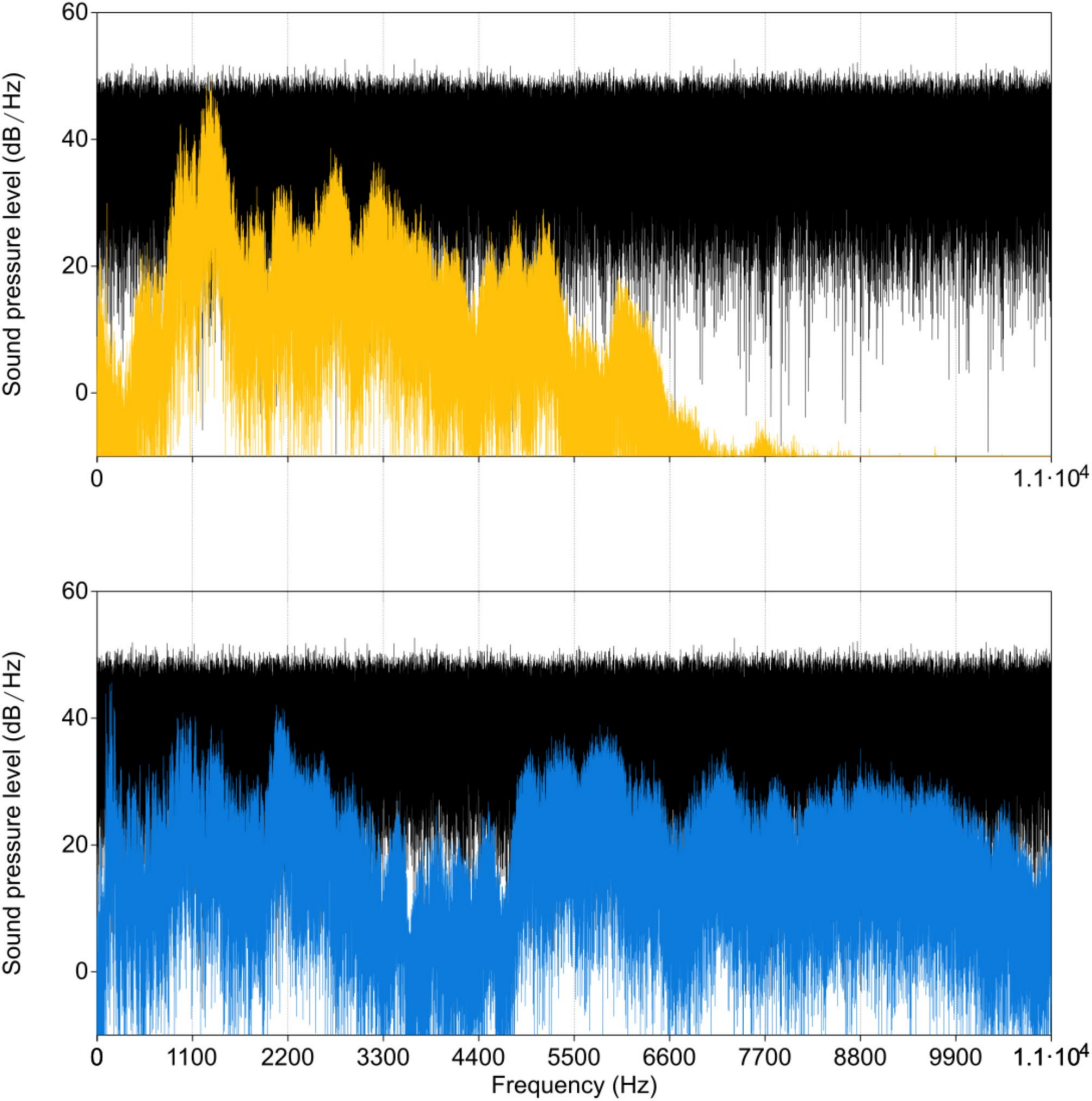
Comparison of the frequency spectrum of the button (yellow, above) and speaker (blue, below) playbacks of white noise (black).

This comparison revealed that the speaker produced a relatively even frequency response with some loss of power between 3 and 5000 Hz compared to the original sound, the button produced a significant loss of power in the same range, but also in the lower frequencies between 0 and 1000 Hz and a dramatic drop above 5000 Hz frequencies (Fig. 1). This suggests that the button playbacks lose a significant amount of spectral information in frequency ranges used in human speech and dog hearing.

## Study 1

### Introduction

A previous study using tape-recorded action cues found that dogs struggled to respond to recorded trained verbal cues, possibly due to differences in frequency composition compared to spoken words^7^. Our analysis of the devices’ frequency outputs also revealed different levels of degradation between devices, with the button sound being more heavily degraded (see above). In this study, we investigated how dogs’ responses differ between devices compared to live verbal cues and expected the degradation to affect dogs’ recognition and response to the trained verbal cues. If the level of degradation matters, we predicted a negative association between degradation and recognition so that the device with heavier degradation (the button device) should more heavily affect recognition and response in dogs than the less degrading device (the JBL speaker).

### Methods

All experiments were performed in accordance with relevant guidelines and regulations and adhered to the ARRIVE guidelines. Ethical permission for conducting this study was obtained from The Institutional Committee of Eötvös Loránd University (N. PE/EA/691-5/2019). All owners gave informed consent for participation and publication, including videos and photographs.

#### Subjects and location

Seventeen typical (T) dogs (4 males, 13 females, mean (± SD) age = 7.1 ± 3.6 years old; 6 Labrador retrievers, 3 Toy poodles, 2 Golden retrievers, 1 mixed breed, 1 Dalmatian, 1 English cocker spaniel, 1 Shetland sheepdog, 1 Welsh corgi, and 1 West highland white terrier) participated. Participants were recruited through social media. The criterion for selecting the dogs was their previous successful learning of three action verbal cues (assessed as described below). Tests were conducted in Japan, either online (*N* = 4) from a room familiar to the dogs, or in person at a familiar designated facility with the experimenter present, depending on the availability of the owners.

#### Experimental setup

##### Preliminary training

Prior to the start of the experiment, participants had trained their dogs to successfully perform three object-free actions of their choice. The selection of these three actions varied among participants, resulting in a total of seven distinct actions across all participants. The trained actions included raising both paws in the air, lying down, spinning in a circle, lowering the head, raising a paw, walking backward, and standing on all four legs (see Supplementary Table S2). They were body movements performed starting from a consistent position of the dog which was unvaried across actions. When delivering these verbal action cues, such as “Bear!”, “Down!”, and “Spin!”, the owner remained seated in a chair, looking straight ahead, without moving the head or the body and with the arms resting on the lap to ensure that no gestural cues were given. The dog and owner faced each other approximately 1 m apart, with the dog off-leash. In each trial, before asking the dog to perform the given action, the owner always directed the dog in the same starting position (seated or standing, based on the trained starting position of each dog during preliminary training), using cues known by the dog. To be recruited in the experiment, dogs had to show proficiency in at least 5 out of 6 trials across two separate sessions in which each action was requested two times (6 trials in total), in a random order determined by the Experimenter. The owner and dog were positioned as described above during the training.

#### Recording of the stimuli

The owners recorded the three verbal cues, one at a time. Each cue was simultaneously recorded to both smartphone and button device, with one button assigned per action cue. This parallel recording ensured that the same audio was recorded on both devices so that they captured the same utterance in the same conditions, helping to maintain consistency. The recordings were conducted in the dogs’ absence to ensure they did not hear the cues outside of the experimental context. Owners were instructed to wait for one second after the devices began recording before speaking the word to be recorded. This precaution considers potential audio latency caused by Bluetooth technology during playback. Owners then practiced synchronizing mouth movements with the sound emitted by the devices, always in the absence of the dogs. Each of the three recorded buttons was then labeled with sticker notes, clearly indicating which button corresponded to the specific verbal cue. On the smartphones, the recorded sounds were named according to the respective action cues and saved in WAV format on The Voice Record Pro app to facilitate identification. The volume of the sound on the buttons and the speaker were adjusted by listening, with the aim of achieving consistent playback loudness.

#### Test procedure

During the test, the owner took the seated position as described above, facing the dog and looking straight ahead. The dog was in front of the owner, in the trained starting position (i.e., sitting or standing, depending on the trained position for each dog). When the tests were conducted remotely, a camera and an online conference software (Zoom Video Communications, Inc.) were used, enabling the experimenter to see the owner and the dog and to communicate with the owner to provide instructions throughout the experiment, as well as to record the test. The camera was positioned to enable the Experimenter to observe both the owner’s and the dog’s actions from the side. When the tests were conducted in person, the camera used to record the sessions was positioned approximately 3 m away from the owner and the dog to capture both from the side.

In each of the five experimental conditions outlined below, the experimenter directed the owner about which action to request in each trial by verbally using a predetermined agreed term, unfamiliar to the dog, for each specific action, such as using “floor” instead of “down”. This precaution ensured that the dog did not hear the familiar spoken action cues from the Experimenter. The actions requested in each trial were predetermined in a semi-random order, preventing requests for the same action in more than two consecutive trials. In every trial, the owner first ensured that the dog was paying attention to him/her, using cues known by the dog, then announced the action cues while staying seated and looking ahead as described above. If the dog performed the requested action, it received praise and a food reward. When the dog made a mistake or did not initiate any movement within 4 s, the owner was directed by the Experimenter to move on to the next trial.

Each of the 3 trained actions was requested in 5 trials across a testing session of 15 trials. Five testing sessions of 15 trials each were conducted across one to two days (depending on the availability of the owners). Breaks were taken between sessions as needed to ensure the well-being and motivation of the dog. In each session, the dogs were tested in 5 different conditions for each of the 3 actions, presented in a semi-randomized order, with no more than two consecutive repetitions of the same condition.

#### Conditions

**V** *Verbal*: The owner spoke the action cues as in the preliminary training.

**B** *Button*: The owner held the button on his/her chest with the speaker side oriented toward the dog and pressed it without moving his/her mouth.

**B + M** *Button + Mouth*: The owner held the button on his/her chest and moved his/her mouth in synchronization as if saying the word while pressing it.

**Sp** *Speaker*: The owner wore a neck strap with the speaker hanging in front of his/her chest, oriented toward the dog while holding the phone, and tapped the play icon without moving his/her mouth.

**Sp + M** *Speaker + Mouth*: The owner wore a neck strap with the speaker hanging in front of his/her chest, oriented toward a dog while holding the phone, and moved his/her mouth in synchrony as if saying the word while tapping the play icon on the app.

For examples of each condition, see Supplementary Video S1 https://youtu.be/eSuj3nCrDss.

### Statistical analysis

Analyses were conducted using the R statistical language (version 4.4.0; R Core Team, 2024)^33^. We compared dogs’ (*N* = 17) success in performing the requested action among the different conditions using binomial Generalized Linear Mixed Models (GLMM; R package “lme4”^34^). Initial models included the fixed effects of condition (factor with five levels), age, trial, and action (factor with seven levels; see Experimental Setup for details about the seven actions), with dog name (ID) as a random effect. We examined condition and trial interactions to assess whether the effect of the condition manifested training success over time. The effects of explanatory variables were analyzed by likelihood ratio tests (LRT), and we provide χ^2^ and p-values of LRT of models when including and excluding the explanatory variable. We also provide pairwise comparisons of estimated marginal means from the final model reached by stepwise model selection.

## Results

**Fig. 2:**
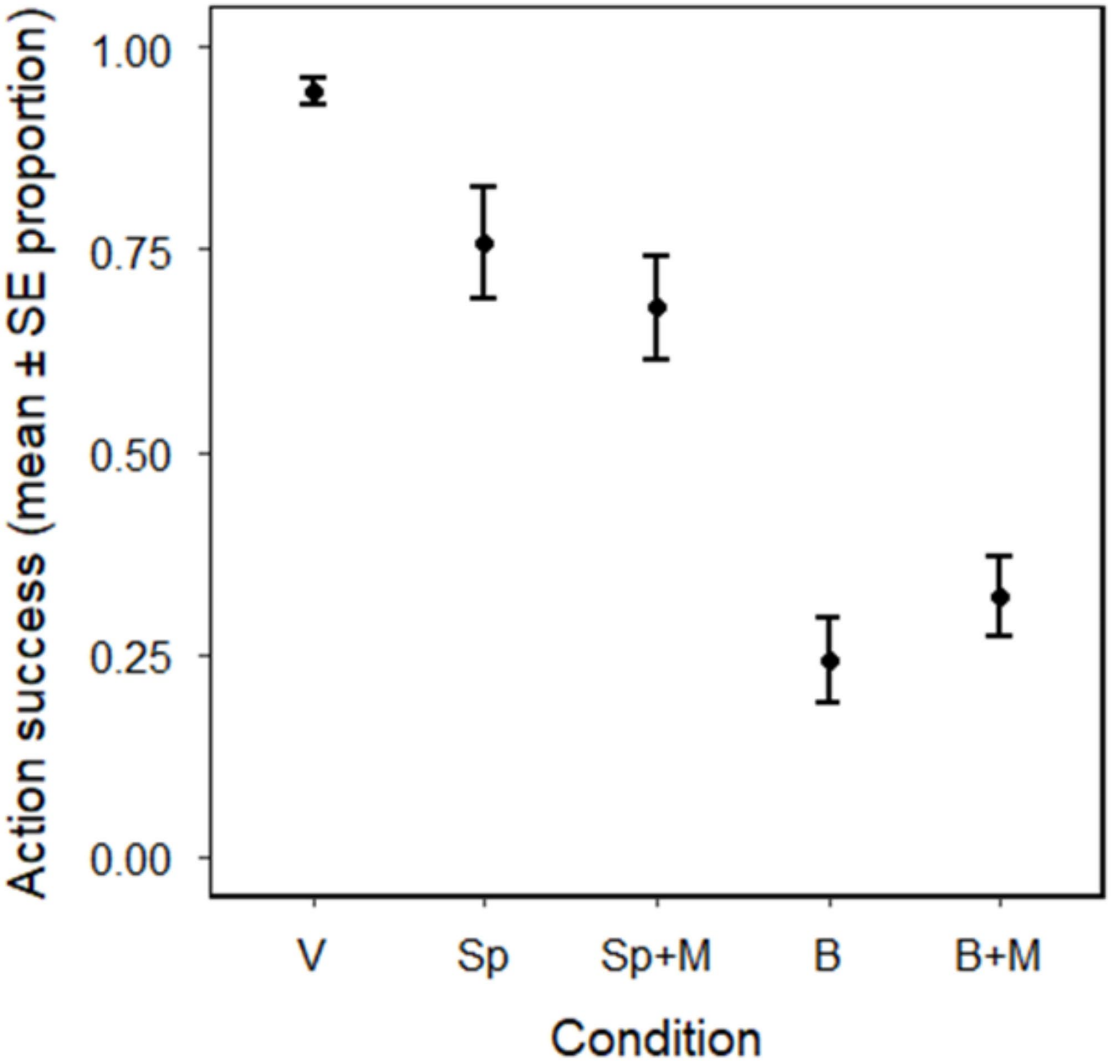
Action success in dogs when the cue is delivered verbally and by different devices. The error plot shows the proportion of successful responses by dogs to follow the 3 trained action cues in the different experimental conditions: verbal (V), speaker (Sp), speaker with mouth (Sp + M), button (B), and button with mouth (B + M).

The results showed significant differences in dogs’ success in performing the requested actions based on the condition, with performance being the worst when the button device was used (binomial GLMM of action success, LRT of condition: χ^2^_4_ = 469.4, *P* < 0.001). The post-hoc pairwise comparisons of estimated means revealed that apart from the two condition pairs with and without mouth movements (speaker vs. speaker with mouth and button vs. button with mouth), performance was significantly different in all possible comparisons of conditions (Table 1). The success was the highest in the verbal condition, followed by lower performance in the speaker and speaker with mouth conditions. The worst performance was in the button and button with mouth conditions (Fig. 2; Table 1).

Besides condition, the final model also included action (LRT of action: χ^2^_6_ = 32.9, *P* < 0.001), as actions were not equally difficult to perform with a significantly lower success when spinning was the requested action (Supplementary Table S3). The other investigated explanatory variables had no effect and were excluded from the final model.

**Table 1:** Pairwise comparisons of estimated marginal means from the binomial generalized linear mixed model of action success.

Contrast	Estimate	SE	z-ratio	*p*-value
V vs. Sp	2.029	0.342	5.930	< 0.001
V vs. Sp + M	2.509	0.340	7.383	< 0.001
V vs. B	4.752	0.356	13.346	< 0.001
V vs. B + M	4.305	0.349	12.335	< 0.001
Sp vs. Sp + M	0.479	0.220	2.176	0.189
Sp vs. B	2.723	0.234	11.628	< 0.001
Sp vs. B + M	2.276	0.225	10.134	< 0.001
Sp + M vs. B	2.244	0.222	10.120	< 0.001
Sp + M vs. B + M	1.796	0.212	8.476	< 0.001
B vs. B + M	-0.447	0.213	-2.103	0.219

## Discussion

The results show that the device used to playback the trained cues significantly affected the dogs’ action success. Dogs performed almost flawlessly in the verbal condition, as expected, given that they were trained to perform the actions upon hearing their respective verbal cues. The dogs’ performance dropped when the cues were played back from the two devices, with a striking drop and the lowest success when the cue was played back from the AIC buttons. This suggests that dogs do not recognize the words when they hear them from this device. We found no differences between device conditions with or without mouth movement, suggesting that seeing the owner’s mouth movement does not affect the dogs’ performance.

## Study 2

### Introduction

The sound analysis we carried out (see above) showed different levels of degradation when human words are played back by the JBL speaker and by the button devices, with a heavier degradation caused by the button device. The results of Study 1 suggest that this level of degradation may compromise typical dogs’ recognition and response to trained verbal cues. Previous studies showed that two GWL dogs could recognize the names of their toys when these were pronounced by persons other than their owners^35^, even when the person was from a different nationality, thus speaking with a different accent^29^. This suggests that GWL dogs may be able to recognize the learned words even with some audible differences. However, the level of audible differences caused by degradation in recorded words that they can tolerate without a significant loss in recognition is not known. In this study, we investigated GWL dogs’ response to the toy names within their learned vocabulary under similar experimental conditions as in Study 1.

### Methods

#### Subjects and location

Seven Gifted Word Learner (GWL) dogs (3 males, 4 females, mean (± SD) age = 4.7 ± 1.5 years old; 5 Border collies, 1 miniature Australian shepherd, and 1 Blue heeler) and their owners volunteered for this study. Tests were conducted at participants’ homes via online meetings with the Experimenter^30^.

#### Experimental setup

#### Recording of stimuli

Study 2 employed identical equipment of buttons, loudspeakers, and recording apps on the participating owners’ smartphones as those utilized in Study 1. For each GWL dog, 8 named toys were randomly selected from the toys successfully tested in previous studies^36^. The selected toys were allocated randomly into four pairs, each pair to be used in one testing session.

The owners recorded each toy’s name on a button device and smartphone simultaneously, as in Study 1. Recordings were conducted in the mother tongue of each participant as participants belonged to different nationalities: Dutch, English (US, UK), Hungarian, and Portuguese. The recordings were carried out without the dogs’ presence to ensure that the dogs would not anticipate which toy was to be requested. The same precautions to control for audio latency and volume adjustment as in Study 1 were implemented.

#### Test procedure

The experiment was conducted in two separate rooms: a toy room and a starting room in the owners’ homes (see also^29^). Before the testing session, four different pairs of test toys were selected to be used in the tests under different conditions. For each testing session, one pair of the selected toys was placed in the toy room alongside 18 other named toys randomly selected from the dog’s collection. The 20 toys (the selected pair and the 18 other named toys) were randomly scattered on the floor in the toy room. Two camera-enabled devices, such as smartphones or tablets, were placed in each room and individually logged into an online conference platform (Zoom) hosted by the Experimenter (see^29^). This setup allowed the Experimenter to observe the dogs’ behavior in the toy room in real-time while simultaneously communicating with the owner in the starting room. The Experimenter instructed the owner as to which toy he/she should request the dog to fetch during each trial by verbally stating the initial letter of the toy’s name so that the dog could not hear the name as spoken by the Experimenter. The camera device in the toy room was muted to prevent echoing and positioned to ensure all the toys on the floor were visible, allowing the experimenter to monitor the dog’s choice^29^.

In each experimental trial, the owner was instructed by the experimenter to request the dog to retrieve one of the two toys of which the owner had recorded the names. The same five conditions were administered as in Study 1 (V, B, B + M, Sp, Sp + M; see above), semi-randomizing the presentation of the different conditions within each testing session. In each trial, before each button press, app operation, or vocalization, the owner said the sentence he/she typically uttered to request fetching a named toy, for example, “< dog’s name>, go get …!“. The toy’s name was then announced once, using voice, button, or speaker, based on the predetermined condition. If the dog successfully retrieved the correct toy, it received praise and was rewarded with social play using the toy, along with a food reward if this was the typical routine for a given dog. When the dog brought the wrong toy, did not bring any toy, did not initiate any movement or did not return from the toys room within 20 s, the owner was directed by the Experimenter to proceed to the next trial.

In addition to the experimental trials in which the owner asked for the two tested toys, we also interspersed the testing session with five motivational trials, where extra toys familiar to the dog were requested through direct vocal request. This was done to keep the dogs motivated and focused throughout the testing session. In total, four testing sessions, each consisting of 15 trials (10 experimental + 5 motivational), were conducted, with four different pairs of recorded toy names and five of the 18 randomly selected toys from the dog’s collection. Each session included 10 experimental trials whereby the 2 toys were tested under the five conditions in a semi-randomized sequence, ensuring that the same condition and the same toy were not repeated more than twice consecutively. Motivational trials were not included in the statistical analysis. The four test sessions were conducted over 2 to 4 different days, depending on the owner’s availability.

### Statistical analysis

Similar to the analysis of Study 1, we analyzed whether the GWL dogs (*N* = 7) could select and retrieve the requested toy using binomial GLMMs. Initial models included the fixed effects of trial (1–60) and condition (factor with five levels). The interaction between trial and condition was also examined. (For more details on the analysis methods, see Study 1).

## Results

**Fig. 3:**
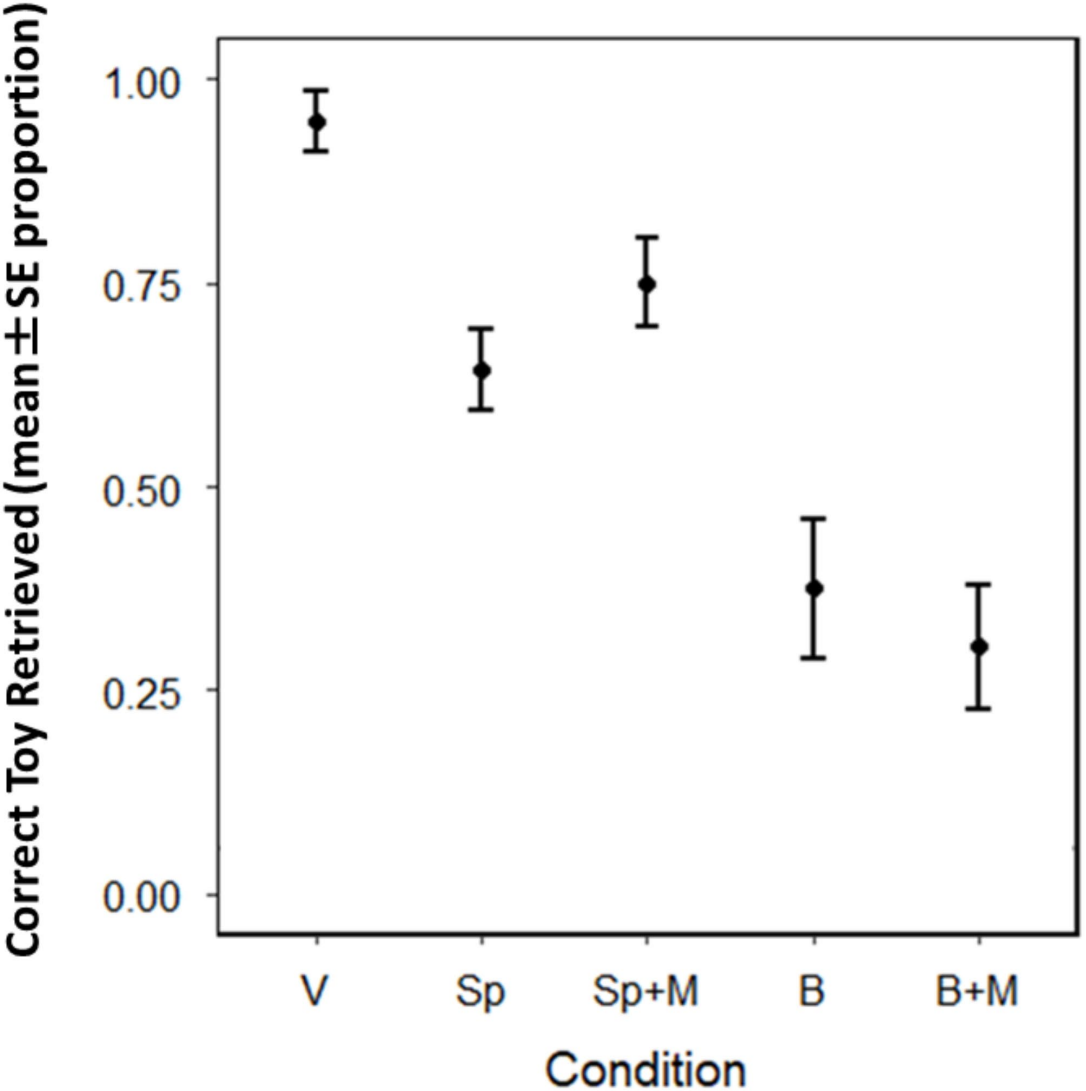
GWL dogs’ success in choosing the requested toy when the toy name is delivered verbally and by different devices. The error plot shows the proportion of successful fetches by GWL dogs when the name of the toy was requested in the different experimental conditions: verbal (V), speaker (Sp), speaker with mouth (Sp + M), button (B), and button with mouth (B + M).

The results showed a similar pattern to that of Study 1; GWL dogs’ success in choosing the named toys was influenced by the experimental condition (binomial GLMM of success, LRT of condition: χ^2^_4_ = 75.84, *P* < 0.001). Like in Study 1, the highest success rate was achieved in the verbal condition, followed by lower performance in the speaker and speaker with mouth conditions, and the worst performance was observed in the button and button with mouth conditions (Fig. 3; Table 2). According to the pairwise comparisons of estimated means, except for the two condition pairs with and without mouth movements (speaker vs. speaker with mouth and button vs. button with mouth), performance was significantly different in all possible comparisons of conditions (Table 2).

**Table 2:** Pairwise comparisons of estimated marginal means from the binomial generalized linear mixed model of success in choosing the correct toy.

Contrast	Estimate	SE	z-ratio	*p*-value
V - Sp	2.342	0.662	3.536	0.004
V - Sp + M	1.811	0.674	2.685	0.056
V - B	3.491	0.665	5.250	< 0.001
V - B + M	3.824	0.673	5.686	< 0.001
Sp - Sp + M	-0.531	0.424	-1.252	0.721
Sp - B	1.149	0.403	2.853	0.035
Sp - B + M	1.482	0.414	3.580	0.003
Sp + M - B	1.680	0.426	3.948	< 0.001
Sp + M - B + M	2.013	0.437	4.611	< 0.001
B - B + M	0.333	0.409	0.814	0.927

## Discussion

The results of GWL dogs show a very similar pattern to those of typical dogs in Study 1, showing that the playbacks from different devices compromise not only typical dogs but also GWL dogs’ recognition and response to words compared to when the words are directly spoken. Although GWL dogs can generalize spoken words with different accents, their lower performance with playback suggests they perceived those recorded words as distinct from live speech. Dogs’ performance using the button devices was remarkably low, with approximately a 70% drop in their success. This study also confirmed that dogs did not rely on visual cues related to mouth movements.

## Study 3

## Introduction

GWL dogs are known to rapidly learn novel toy names during playful interactions in which their owners utter the name of the toy^29^, but it is not known whether they would learn new toy names from a recorded voice presenting some level of degradation. In this study, we investigated whether GWL dogs would learn new toy names from a recorded voice and whether they generalize from the learned sound (the word played back from the device) to when the owners utter it directly. Considering the results of Study 1 and 2 regarding buttons’ heavy degradation and the consequent lack of word recognition and response by the dogs, we used only the configuration of a smartphone and JBL speaker for this study, as they provide better word recognition and response. Given that both the smartphone’s microphone and the used speaker dampen some frequencies, the direct human voice should provide richer spectral information (i.e., the same sounds provided by the speaker and, in addition, those lost in the speaker playback). For this reason, we expected that GWL dogs would be able to generalize the learned sounds, at least to some extent.

### Methods

#### Subjects and location

*N* = 9 Gifted Word Learner (GWL) dogs participated in this study, 6 of which also participated in Study 2 (3 males, 6 females, mean (± SD) age = 4.7 **±** 1.8 years old, 6 Border collies, 1 miniature Australian shepherd, 1 Blue heeler, and 1 German shepherd).

#### Experimental setup

#### Recording of stimuli

The owners were required to select names for the novel toys that were not similar to other toy names already familiar to their dog. Before the training started, the owners recorded the names of the novel toys on the smartphone using the same method as previously described. All other experimental conditions, including the test location, equipment, precautions, and two-room setup, were consistent with those described in Study 2 (for details, see Study 2).

#### Test procedure

This study included two phases: the treatment phase and the control phase. In both phases, GWL dog owners were tasked with teaching their dogs the names of 4 novel toys in 2 weeks. The test was carried out 2 weeks after the start of the training in each phase.

In the treatment phase, owners used a JBL speaker to play back recordings of toy names to teach the names of four novel toys to their dogs. During the training, the toy names were never uttered by the owner, who, instead, used the recorded voice played through the speaker, for example, “Look! It’s < replay of the toy name>”. In the control phase, the owners directly spoke the toy names to teach the names of a different set of four novel toys, following their usual verbal teaching methods. (See Supplementary Video S2 https://youtu.be/CfmVppJ6AJE for an example). The control phase was aimed at ensuring that the tested GWL dogs were able to learn the names of 4 novel toys in two weeks. The only difference between the two phases was the method of delivering the toy names during the training period.

For both phases, the novel toy names were taught during the typical playful interactions in which GWL dogs learn toys^29^. The owners were requested to dedicate the necessary time to teach toy names as they usually do with their dogs. According to the owners, an average of 10 (mean ± 1.40 SD) minutes per day was spent teaching. The order of administration of the two phases was randomized across dogs.

In both phases, we used the same two-room setup and camera arrangement as in Study 2. In the tests, dogs were required to choose from a total of 10 toys: the four novel toys learned during the training and six familiar named toys randomly chosen from the dogs’ pre-existing toy collection.

#### Treatment phase test

In each trial, the owner was instructed to request the dog to retrieve one of the four new toys taught during the training from the toy room. Two conditions were administered: the speaker condition (Sp) to test training outcomes and the verbal condition (V) to test generalization. In both conditions, first, the owner pronounced the typical sentence used to request a named toy (e.g., “Go get …”). Then, in the speaker condition, the owner played back the recorded toy name through the JBL speaker. In the verbal condition, the owner said the name.

A total of 24 trials were conducted: 12 for each condition, consisting of 3 trials for each of the four toys in a semi-randomized order, ensuring that the same condition and same toy were not repeated in two consecutive trials. Additionally, 6 motivational trials with toys from the dogs’ collection were included to keep the dogs motivated. The motivational trials were not included in the data analysis. During the test, the owners were instructed to return the toys fetched by the dogs to the toy room as needed to ensure that at least eight toys were always present for the dogs to choose from. Among these, at least three out of the four novel toys were always available for the dog to choose from. The experiment was conducted over one to two days per dog, depending on the availability of the owners.

#### Control phase test

Using the same setup and procedure as in the Treatment phase test, only the verbal condition was tested, whereby the owner verbally requested the toys. A total of 12 trials were conducted, 3 trials for each of the four toys, along with 6 additional motivational trials, as in the Treatment phase test.

### Statistical analysis

The dogs’ correct or incorrect choice of the four toys learned in each experiment was recorded during the test. In line with the above, we used binominal GLMMs to analyze whether the GWL dogs (*N* = 9) could select and retrieve the requested toy. We applied two approaches. In the first approach, we focused on training outcomes by including the subset of tests with speaker conditions from the treatment phase and the verbal trials of the control phase. Therefore, in this analysis, 12 treatment phase and 12 control phase trials were compared, so the difference between them was the way dogs were trained and tested either using the speaker (in treatment phase trials) or using verbal request (in control phase trials). Initial models included the fixed effects of trial (1–12) and phase (factor with 2 levels: control vs. treatment). The interaction between trial and phase was also examined.

In the second approach, we focused on the generalization of the speaker training by including treatment phase trials only. Using binomial GLMMs, we compared the responses of dogs between the two conditions, i.e., when tested by the speaker (12 trials) or when tested verbally (12 trials). Initial models included the fixed effects of trial (1–12) and condition (factor with 2 levels: speaker vs. verbal), as well as the interaction between trial and condition. (For more details on the analysis methods, see Study 1).

## Results

**Fig. 4:**
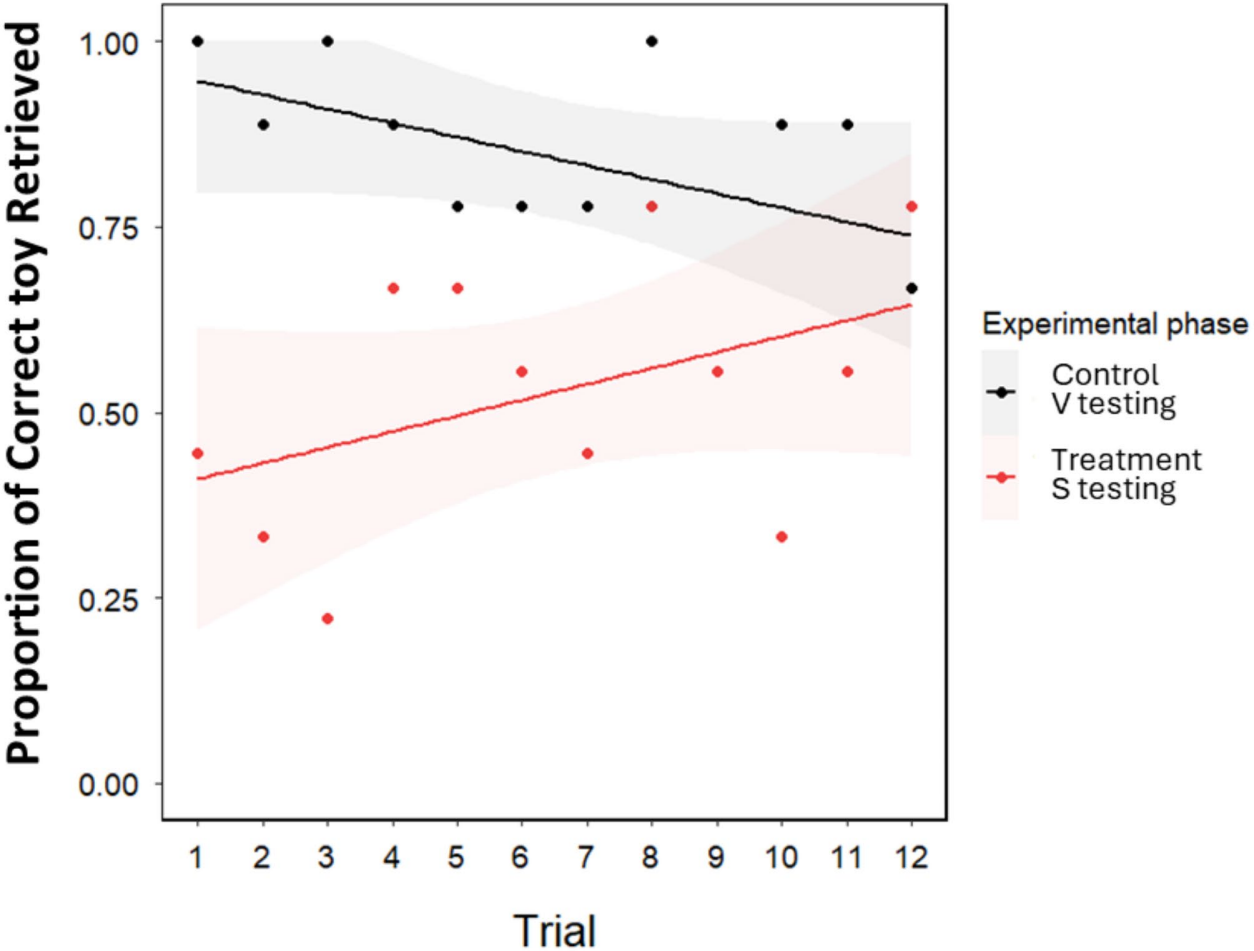
GWL dogs’ success in choosing the requested toy over 12 experimental trials when the toy name was either trained and then tested verbally (control phase) or using a speaker (treatment phase).

The analysis of training outcomes showed a significant effect of the device used for teaching toy names on GWL dogs’ responses (binomial GLMM of success, LRT of experimental phase: χ21 = 29.77, *P* < 0.001; Fig. 4). This was due to the significantly lower performance of the dogs when trained with the speaker compared to when they were trained verbally (mean ± SE of success, speaker vs. verbal training/testing: 0.53 ± 0.05 vs. 0.84 ± 0.04). In addition, this analysis also revealed a trial-specific effect of training, with a decreasing trend of success in the control experiment and an increasing trend in the treatment experiment (LRT of trial x experimental phase: χ21 = 6.96, *P* = 0.008; Fig. 4).

The analysis of generalizability revealed no significant effect of the testing condition, although we found a trend-like, weak effect with a somewhat higher success with verbal testing as opposed to testing with the speaker (LRT of experimental condition: χ21 = 3.62, *P* = 0.057; mean ± SE of success, speaker vs. verbal testing: 0.53 ± 0.07 vs. 0.65 ± 0.08).

## Discussion

The results indicate that GWL dogs learned new toy names through playbacks via a speaker, yet their performance was approximately 30% lower than verbal learning. Interestingly, when tested for generalization, the dogs performed slightly better with direct verbal testing, even though during the training, they were exposed only to recorded toy names, suggesting they not only could transfer their learning from playbacks with some degradation to the direct verbal utterance of the original human speaker, but that the live human voice facilitated their performance. This highlights their flexibility in auditory processing of human speech and may reflect the naturalness or familiarity of live human speech or the seamless flow of direct verbal communication, which aligns more closely with the dogs’ natural interactions with humans. Additionally, the trend of increasing success rates during the treatment phase with the speaker condition suggests that GWL dogs can adapt to this modality, demonstrating their exceptional vocabulary learning ability (e.g^30,37^). It is important to note that these findings should not be directly generalized to typical dogs, as they were shown to struggle to learn object names in previous studies^30,38^.

## General discussion

This study demonstrates that the sound degradation caused by different devices used to deliver audio cues significantly affects dogs’ success in performing trained actions on verbal cues (Study 1) and retrieving specific toys based on toy names (Study 2), thus revealing that dogs’ recognition of learned human words depends on the quality of the played back sounds. In both experiments, hearing the words from the AIC buttons (with or without mouth movement of the owner) resulted in the poorest performance, suggesting that dogs do not recognize human words when played back from this device, most likely due to the critically attenuated frequency components. The performance difference of the dogs between conditions followed a consistent pattern across Study 1 and Study 2, despite the different tasks and the different subjects tested (typical vs. GWL dogs). In both studies, the direct verbal cues led to the highest success, followed by cues emitted by the speaker device, and the lowest success was always observed with cues emitted by the button devices.

Our findings align with human speech recognition studies^2,3^ showing that listeners can recognize speech with degradation to some extent but struggle as degradation becomes more severe. The results also align with previous research using different types of speech degradation to test dogs’ recognition of their names^5,6^: in the previous studies, and also in the present one, the heavier the degradation of human words, the more dogs struggle to recognize those. Similarly, dogs’ best performance in the verbal conditions is in line with previous research showing that dogs’ performance decreases with the use of recorded cues^12,13^.

As expected, due to the training, dogs performed nearly at the ceiling in the voice condition. The performance in the button condition was significantly worse than the performance in the speaker condition, most likely due to differences in degradation levels at the lower frequencies. A comparative analysis of sonograms of recorded sounds from the devices used in this study indicates that the playbacks previously recorded by the audio button devices filter out more frequencies below 1000 Hz than sounds played through JBL loudspeakers, including the fundamental frequency and lower formants. Fundamental frequencies are critical for dogs to process human speech, as they serve as a key element for voice recognition^15^. The absence of these essential frequencies in button-generated sounds likely results in dogs not recognizing (and therefore not responding to) the conveyed words. Thus, the filtering out of crucial acoustic components likely explains the extremely low performance observed in the button condition in Studies 1 and 2.

In citizen science, participants often use consumer-grade devices to collect audio data. A study compared the quality of recordings of bird songs collected by citizen scientists using smartphones with those recorded using professional equipment and found that smartphone recordings were sufficient for broad, general evaluations, such as identifying species presence or song types, but they lacked the precision needed for detailed acoustic analysis^39^. In our study, smartphone-recorded human words played back with a commercially available speaker resulted in a poorer recognition and response to the words compared to when those were directly spoken. However, the smartphone and speaker configuration allowed dogs to perform significantly better than the button devices. The extremely poor performance in the button conditions in Studies 1 and 2 shows that when the degradation of the recorded sound exceeds their capacity, dogs’ recognition and response to those sounds are too heavily affected. Thus, when using recordings, preserving key elements of the human voice, such as fundamental frequency, is crucial for dogs to recognize and respond to the played-back words, and we suggest that citizen science studies should carefully evaluate the balance between affordability and sound quality, if recognition of recorded sounds is needed.

We did not find differences in dogs’ performance between the conditions adding or not mouth movement to the speaker and button conditions. The consistency across studies in these findings suggests that dogs do not rely on the owner’s mouth movements to recognize the different cues. In Study 2, the contrast between “Vocal” and “Speaker + Mouth” did not reach statistical significance. A possible explanation is that GWL dogs may show indifference to this contrast, potentially due to their extensive use of a multitude of verbal labels, making relatively small sound degradations less impactful.

Study 3 highlights the flexibility of GWL dogs in auditory learning, as they were able to generalize somewhat degraded recordings emitted via the JBL speaker to the original human live utterance. However, their lower performance with playbacks emphasizes the critical role of sound quality – and potentially of a smoother learning procedure, more in line with the natural interactions between humans and dogs, in facilitating learning. This finding also aligns with the pattern observed in Studies 1 and 2, where sound degradation consistently hindered dogs’ recognition and response to human speech. Additionally, similar to how the human brain processes live and recorded music differently^40^, it is possible that dogs may have processed live communication and recorded voice in distinct ways. The need to alternate between live and recorded speech during the experiment might have disrupted the dogs’ natural processing of human speech, potentially contributing to the differences in performance between live and recorded cues. The better performance in the generalized live speech condition compared to the trained playback condition has yet to be fully understood. This raises questions about the role of natural spectral properties in human speech processing for dogs. Future studies should explore the impact of acoustic richness, which may provide important information for dogs’ learning.

Given that research often relies on human-recorded voices^10,12–14,19^, the methodological significance of our study lies in its potential to provide fundamental information on how dogs’ recognition of recorded words is affected by using equipment of varying quality of recording and playback. Our results challenge the notion that dogs recognize and appropriately respond to recorded words in the same way they do to spoken words by highlighting the crucial role of audio quality. Thereby, our results suggest that verifying the frequency spectrum of the playback equipment is necessary to ensure sufficient sound quality for dogs to recognize the played-back sounds.

One potential concern in our study is the variation in recording quality between different smartphones, as commercially available devices feature microphones and speakers with varying specifications, along with file compression, that may potentially degrade the signal; however, our preliminary analysis suggests these differences are minor, with results showing low variance in dogs’ performance, indicating that potential degradations from different mobile devices used for recording were minimal.

Based on our findings, further research should systematically explore which frequency ranges dogs rely on to recognize and respond to words. Understanding this could help in optimizing playback equipment designed for dogs, ensuring they meet their audio perceptual capacity.

## Summary

In conclusion, the varying levels of degradation of human speech provided by different digital devices to convey learned words significantly influence dogs’ success in recognizing the words and thus responding, compared to direct speech of object labels or actions. Whereas there are many commercial products available for citizen science studies, such as AIC button devices, our results reveal that, due to the degradation of the sound emitted by these devices, dogs struggle to recognize the recorded words when the sound quality is too heavily compromised. The study also reveals that using digital devices with less audio degradation results in higher success rates of word recognition and response.

## Electronic supplementary material

Below is the link to the electronic supplementary material.

Supplementary Material 1

## Data Availability

All data generated or analyzed during this study will be provided upon reasonable request. For inquiries, contact: fumihigaki2016@gmail.com.
