# Supplementary material for: Sound quality impacts dogs’ ability to recognize and respond to playback words
Source: Sci Rep. 2025 Apr 28;15:14175. doi: 10.1038/s41598-025-96824-8 (PMC12037911; doi:10.1038/s41598-025-96824-8)
Supplement: Supplementary file 1 — Supplementary Material 1 [file 41598_2025_96824_MOESM1_ESM.docx]

Supplementary material for

**Sound quality impacts dogs’ ability to recognize and respond to playback words**

Fumi Higaki^1*^, Tamás Faragó^2^, Ákos Pogány^2^, Ádám Miklósi^1,3,4^, Claudia Fugazza^1,3^

^1^ Department of Ethology, Eötvös Loránd University, Pázmány P. s 1c, 6th Floor, 1117 Budapest, Hungary

^2^ BARKS Lab, Department of Ethology, Eötvös Loránd University, Budapest, Hungary

^3^ ELTE-HUN REN NAP Comparative Ethology Research Group, Budapest, Hungary

^4^ MTA-ELTE Comparative Ethology Research Group, Budapest, Hungary

**Table S1** Audio files used for devices’ frequency comparison

| Audio files (wav) | Link |
| --- | --- |
| Original noise | [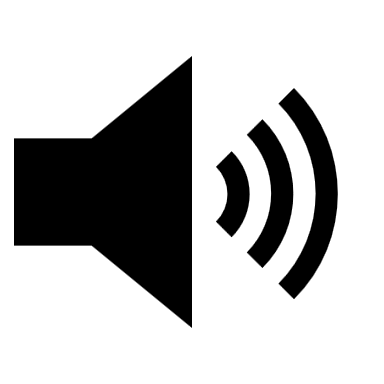](https://drive.google.com/file/d/131OI2r3xdQaF7wTqmU3UaUT2aUyA7m9F/view?usp=sharing) |
| Button playback | [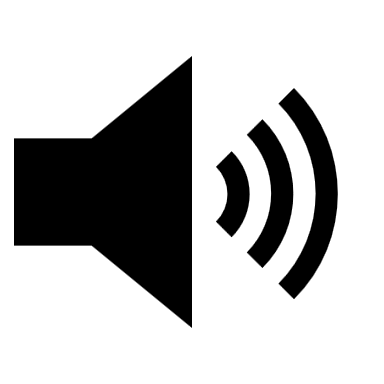](https://drive.google.com/file/d/133wGH2DJylwOFEAtcpAyQhp47Wdf6f8S/view?usp=drive_link) |
| Speaker playback | [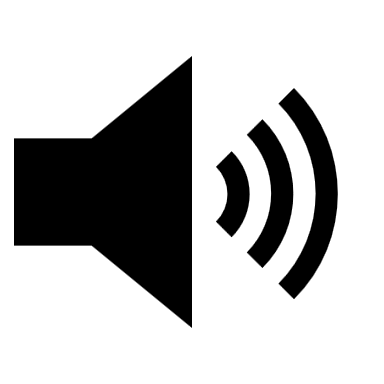](https://drive.google.com/file/d/13A34EBzqvsN2WL63FWexlYiy-gVklXwg/view?usp=sharing) |

**Table S2** Seven actions included in Study1.

**Table S3** Pairwise comparisons of estimated marginal means from the binomial Generalized Linear Mixed Model across actions in Study 1.

Video S1: Example of each test condition in Study 1

<https://youtu.be/eSuj3nCrDss>

Video S2: Example of training in each phase in Study 3

<https://youtu.be/CfmVppJ6AJE>
